# Recent Advances in Inorganic Nanomaterials Synthesis Using Sonochemistry: A Comprehensive Review on Iron Oxide, Gold and Iron Oxide Coated Gold Nanoparticles

**DOI:** 10.3390/molecules26092453

**Published:** 2021-04-22

**Authors:** Mohammed Ali Dheyab, Azlan Abdul Aziz, Mahmood S. Jameel

**Affiliations:** 1 Nano-Biotechnology Research and Innovation (NanoBRI), Institute for Research in Molecular Medicine (INFORMM), Universiti Sains Malaysia, Pulau Pinang 11800, Malaysia; shokryy8844@gmail.com; 2 Nano-Optoelectronics Research and Technology Lab (NORLab), School of Physics, Universiti Sains Malaysia, Pulau Pinang 11800, Malaysia

**Keywords:** sonochemistry, nanomaterials synthesis, iron oxide gold nanoparticle, shape control

## Abstract

Sonochemistry uses ultrasound to improve or modify chemical reactions. Sonochemistry occurs when the ultrasound causes chemical effects on the reaction system, such as the formation of free radicals, that intensify the reaction. Many studies have investigated the synthesis of nanomaterials by the sonochemical method, but there is still very limited information on the detailed characterization of these physicochemical and morphological nanoparticles. In this comprehensive review, recent advances in the sonochemical synthesis of nanomaterials based on iron oxide nanoparticles (Fe_3_O_4_NP), gold nanoparticles (AuNP) and iron oxide-coated gold nanoparticles (Fe_3_O_4_@Au NP) are discussed. These materials are the most studied materials for various applications, such as medical and commercial uses. This review will: (1) address the simple processing and observations on the principles of sonochemistry as a starting point for understanding the fundamental mechanisms, (2) summarize and review the most relevant publications and (3) describe the typical shape of the products provided in sonochemistry. All in all, this review’s main outcome will provide a comprehensive overview of the available literature knowledge that promotes and encourages future sonochemical work.

## 1. Introduction

Reducing the material’s size to the nanoscale limits the electrons inside to a small space that changes its physicochemical properties [1]. Such an expedited transition has given rise to many new applications while enhancing existing ones. The idea of synthesizing nanomaterials with the appropriate morphology and applicable properties has therefore stimulated great interest in this technological evolution. Through simple solution-based approaches such as hydrothermal, solvothermal and sonochemistry to cutting-edge approaches as ablation, epitaxy and lithography, the diversity of nanomaterial synthesis techniques can be exploited to monitor their development. Sonochemistry provides a special crystallinity control that enables the preparation of amorphous metals, metal alloys and metal oxide [2]. Compared to most approaches, sonochemical is very economical, allowing individual enthusiasts and researchers to experience and try out ideas [3]. It has grown to prominence with a rise in interest in material processing and engineering over the last 30–40 years. Nonetheless, the study of chemical solutions and chemical reactions with the use of sonochemical dates back to the early 20th century [4]. Physical methods such as vapor deposition, plasma glow discharge and gas-phase sputtering are prohibitively costly, whereas chemical methods such as electrolysis, photochemical synthesis and electroreduction yield greater nanoparticle sizes and have problems with mass processing. A third technique, such as the sonochemical process, solved some of the drawbacks described above by generating nanoparticles of smaller sizes [5]. The microwave method requires high temperatures during the synthesis [6,7,8]. The high temperature will lead to too-high reaction kinetics. It is impossible to control the growth step of the crystallization process in reactions with fast kinetics; on the other hand, an explanation for this is that cherry extract is a reducing agent that is rich in ascorbic acid [9], and this acid becomes slightly unstable at higher temperatures [10] and leads to a poor reduction process and uncontrolled and fast aggregation. Sonochemical syntheses takes less than an hour to produce compared to the solvothermal method’s 48-h requirement. Furthermore, the particles generated by sonochemical processes are smaller than any of those generated by conventional synthesis [11].

Zhanfeng et al. [12] analyzed the most recent advances in the field of ultrasound-mediated effects or processes in catalysis, focusing on the production of catalyst materials and sonochemical uses in catalytic processes. Jun et al. [13] clearly present the fundamental concepts of ultrasound irradiation, such as mechanical and physical effects, sonochemical effects and acoustic cavitation, and it hereby summarizes sonochemical catalysis for the manufacturing of nanostructured and microstructured inorganic materials, including plastics, inorganic composites, metal compounds and alloys. Another study by Hangxun et al. [14] showed how the physical and chemical effects of high-intensity ultrasound could be used to prepare or alter several nanostructured materials. However, there is limited information on the detailed characteristics of nanoparticles’ physicochemical and morphological features obtained by intensive (sonochemistry) technologies.

Our goal is to provide a comprehensive review, collecting together all relevant knowledge to better understand nanomaterial synthesis using the sonochemical method, focusing on Fe_3_O_4_ NPs, AuNPs and Fe_3_O_4_@AuNPs and their contributions to different fields. Sonochemical originates from the intense transient conditions produced by ultrasounds, which create unique hot spots that can reach heating and cooling rates of up of 10^10^ K s^−1^, pressures above 1000 atmospheres and temperatures of about 5000 K [15,16]. These conditions vary from other traditional synthetic methods such as flame pyrolysis, wet chemistry, photochemistry and hydrothermal synthesis [17,18]. Ultrasonic waves passing through typical liquids create low- and high-pressure regions based on periodic expansion and compression [19]. This shift in pressure marks the beginning of sonochemistry, so this precedes the critical phase of acoustic cavitation, i.e., the formation, growth and collapse of the bubble. The process of the bubble growth and compression proceeds until the external pressure prevails and the bubble implodes. These conditions can lead to abnormal chemical and physical changes and promote a particular reaction between molecules and atoms to create a special category of materials [20]. However, the utility of the sonochemical process lies in the fact that the radicals and ions inside the bubble emanate from the chemical solutions; thus, suitable chemicals will help to customize the overall procedure. These conditions permit the sonochemical production of different nanomaterials. The sonochemical method has been employed to synthesize a variety of nanomaterials other than noble metals with a variety of structures. Among these materials are CdS NPs [21], Ag_2_Se NPs [22], AgNPs [23], Pt NPs [24] and ZnO NPs [25]; transition metal oxides [26]; silicon oxide nanocrystals [27]; carbon-based materials [28] and metal composites [29].

This review will emphasize the recent studies on using a sonochemical method for nanostructured material synthesis and be arranged according to the mechanisms whereby ultrasounds can be employed to synthesize nanomaterials. In addition, this review will also summarize the most relevant publications on the synthesis of nanomaterials focusing on Fe_3_O_4_, Au and Fe_3_O_4_@Au NPs, as well as describing the typical shapes of the products produced.

## 2. Sonochemical Formation of Fe_3_O_4_NPs, AuNPs and Fe_3_O_4_NPs@AuNPs

The chemical reaction driven via extreme ultrasound waves was strong enough to create cavitation, dissolution, oxidation, hydrolysis and decomposition [30]. Ultrasonic irradiation through aqueous liquids induces free radicals of OH. and H. radicals [31]. These radicals may recombine to return to their original form or combine to generate H_2_O_2_ and H_2_, and these resulting strong oxidants and reductants, in turn, are employed during different reactions of the sonochemical in the aqueous solutions [17].

(1) $$H_{2}O\to \;H^{.}+OH$$

(2) $$OH+OH\;\to \;H_{2}O_{2}\;$$

(3) $$H^{.}+H^{.}\;\to \;H_{2}$$

For Fe_3_O_4_NPs,

(4) $$FeSO_{4}\to \;Fe^{2+}+{\left(SO_{4}\right)}^{2-}$$

(5) $$2Fe^{2+}+H_{2}O_{2}\;\to \;2Fe^{3+}+2OH^{-}$$

(6) $$Fe^{2+}+Fe^{2+}+8OH^{-}\;\to \;Fe_{3}O_{4}+4H_{2}O$$

For AuNPs, the formation of the AuNPs takes into account the fact that free radical species are produced by the ultrasonic irradiation of water molecules (Equation (7)).

(7) $$\begin{array}{c} Na_{3}C_{6}H_{5}O_{7}\to \;Na_{2}C_{5}H_{4}O_{5}+CO_{2}+Na^{+}+H^{+}\;2e\\ Au^{+}+2e\;\to \;Au^{0}\; \end{array}$$

The formation of Fe_3_O_4_NPs@AuNPs and the sonochemistry mechanism for coating the Fe_3_O_4_ surface by Au NPs were discussed in detail in our previous work [32]. Briefly, sodium citrate can activate the reaction of the Fe_3_O_4_ surface with carboxylate ions (–COO^−^), which enables the strong coordination of highly –COO^−^ water with the Fe atom of Fe_3_O_4_ NPs. The mechanism that controls the bonding of Au NPs to the Fe_3_O_4_ surface is linked to the microjets and shock waves generated within the vicinity of surfaces of the solid, following the bubble collapse [33]. These microjets initiate the sintering of micrometer-sized metallic particles and drive the NPs in the direction of the Fe_3_O_4_ surface at very high velocities [33]. The jets react with free carboxylate ions (–COO^−^) on the Fe_3_O_4_ surface, resulting in Au nanoshell formation on the Fe_3_O_4_ surface.

## 3. Nanomaterial Ultrasonic Synthesis

Fe_3_O_4_ NPs, AuNPs and Fe_3_O_4_@AuNPs are the main materials of interest due to their biocompatibility, unique superparamagnetic properties, chemical stability, oxidation resistance and optical properties and which are important for fields such as catalysis and biomedical applications [34,35,36]. Ultrasound irradiation has recently been widely used in the synthesis of nanomaterials [37]. As clarified, high-intensity ultrasound results primarily in acoustic cavitation [38], which, in turn, initiates a distinctive interface between energy and matter [39]. This enables a wide range of chemical reactions and the production of a range of exceptional nanostructured materials [17]. Acoustic cavitation dynamics depend on the localized environment, either a uniform liquid or an inhomogeneous interface between a solid and liquid. For homogenous liquids that produce spherical cavities, acoustic cavitation produces implosive bubbles and waves, generating higher pressure with amplitudes above 10 kbar [40]. Conversely, the acoustic cavitation is asymmetric for an inhomogeneous medium and is related to high-speed microjets that influence the solid surface, causing mechanical damage [41]. The collapsing bubble’s potential energy is converted into the microjet’s kinetic energy with speeds of 100 s of m/s. Nonetheless, Doktycz and Suslick announced that solid particles under the collapsing bubble diameter (~150 μm) unable to start a microjet formation after an ultrasonic field of 2 kHz were irradiated [42]. Alternatively, traditional cavitation and shock wave emissions occurred [43].

### 3.1. Fe_3_O_4_NPs

Fe_3_O_4_NPs can easily be synthesized using a sonochemical method via very high pressures and temperatures generated by ultrasonic irradiation by decomposing iron salts and other nanostructures from an inorganic iron precursor [44]. The ultrasonic irradiation generates high temperatures that lead to the formation of Fe_3_O_4_NPs through the decomposition of iron salts [45]. Fe_3_O_4_NPs improved their hydrophilic and monodisperse properties through the ultrasonic irradiation process [46,47]. Many authors have recently reported using a sonochemical way to synthesize Fe_3_O_4_ with promising physicochemical properties, such as a high surface area and high electron storage ability [48,49].

**Figure 1:**
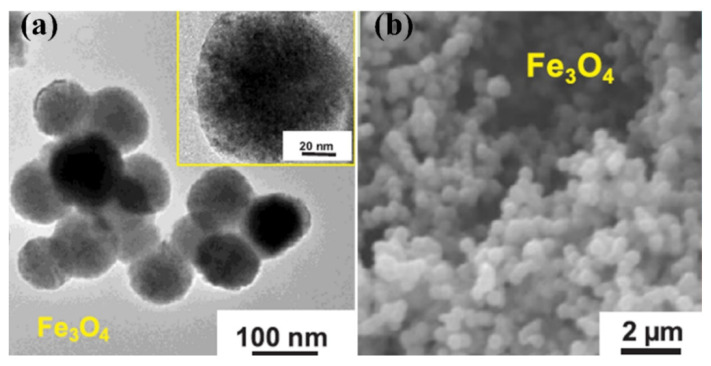
Images of Fe_3_O_4_NPs decorated by GOS synthesis using the sonochemical method (**a**) TEM and SEM (**b**) [51]. Copyright 2019 Elsevier.

Zuzana and Kerstin synthesized Fe_3_O_4_NPs and coated them with dextran as a capping agent [50]. The larger cavitation bubbles at lower frequencies release more energy and create exceptional reaction conditions concerning pressure and temperature, further facilitating the development of the larger magnetite cores. This method has several advantages, such as a high chemical reaction rate, high yields and cost-effective synthesis. Sriram et al. reported a rapid and straightforward fabrication of Fe_3_O_4_NPs decorated by graphene oxide (GOS) by the sonochemical method [51]. This process involves a GOS ultrasound-assisted reduction reaction. Furthermore, the advantages of the modified Fe_3_O_4_NPs decorated by GOS were its reproducibility, repeatability and high stability. Figure 1a,b presented TEM and SEM images of Fe_3_O_4_NPs decorated by GOS. The nanoparticles have a spherical shape with a size of about 96 nm (Table 1).

An ultrasonic-assisted coprecipitation approach of synthesizing magnetite nanoparticles was recorded by Villegas et al. [52]. This approach identified a 16-nm amphoteric crystallite size and provided a superparamagnetic behavior that separated less easily from a solution in 1 min by using only a magnet. Fe_3_O_4_NPs decorated on multi-wall nanotubes (MWCNTs) were successfully produced using a rapid and easy sonochemical process without any chemical treatment on MWCNTs [53]. Fe_3_O_4_@ MWCNTs displayed a uniform, fine nanoparticle distribution in the MWCNTs. Fe_3_O_4_@MWCNTs demonstrated an effective catalytic efficiency after 1 h of treatment with 20-mg/L persulfate. Fe_3_O_4_NPs helped produce sulfate radicals and hydroxyl radicals in the Fe_3_O_4_@ MWCNTs hybrid catalyst, whereas the size of the Fe_3_O_4_ clusters could affect the transmission of electrons for radical production. In addition, the combination of persulfate and Fe_3_O_4_@MWCNTs decreased the remaining cell number to 9.4% within 30 min of treatment using high-frequency and low-intensity ultrasounds. In summary, this method showed that low frequencies of ultrasonic devices were capable of manufacturing Fe_3_O_4_@MWCNTs through a simple and fast method.

Due to the strong influence of the nanoparticle size on the magnetic and structural properties of Fe_3_O_4_NPs, the size selection in proportion to the desired magnetization of these particles is very significant. Boustani et al. successfully demonstrated the synthesis of Fe_3_O_4_NPs by ultrasonic treatment (40 kHz, 150 W) and a novel precipitating agent (ethylenediamine) by the coprecipitation way [54]. It was found that ethylenediamine produces Fe_3_O_4_NPs with a larger size and desired magnetization saturation (Ms). In another study, a single, inexpensive and nontoxic metal salt (FeSO_4_.7H_2_O) reactant was used in aqueous media to synthesize monodisperse Fe_3_O_4_ nanocubes with a uniform particulate size of about 80 nm using the sonochemical method [55]. The magnetic properties of Fe_3_O_4_ nanocubes demonstrated a magnetization of 91 emu/g for the as-synthesized sample at 5 K and of 94.8 emu/g for the sample that was annealed by a vacuum chamber at 600 °C. Due to high magnetization, these nanoparticles can be used for various applications, such as MRI and drug delivery.

### 3.2. AuNPs

Over the past decades, nanomaterials, especially AuNPs, have attracted a great deal of attention from the research community because of their different and unique physical, chemical, photochemical, electronic and optical properties, which also differ from the material properties in their bulk states [56,57,58]. The unique characteristics of AuNPs include high stability and chemical stability, high surface area-to-volume ratios and a surface plasmon resonance (SPR) effect [59,60,61,62]. AuNPs are also biologically unreactive, biocompatible and can be used with pharmaceutical drugs, proteins and enzymes [63]. For this purpose, they can be used in diverse medical applications, such as sensors and biosensors [64], tissue imaging [65], therapeutic agents [66], dentistry [67,68], catalysts [69], diagnosis treatment [70] and drug and gene delivery [71].

**Figure 2:**
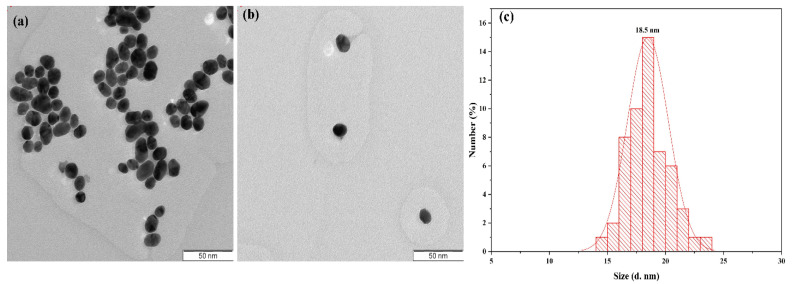
(**a**,**b**) TEM photograph with a scale bar of 50 nm and (**c**) AuNPs with a size distribution of about 18.5 nm synthesized within 5 min at an ultrasonic output power of 17.9 W [80].

Recently, several methods of synthesis AuNPs have been developed to determine the physical and chemical properties of AuNPs by their surface structures, purity, size and morphology [72,73]. These methods are: (i) pyrolysis processes [74], (ii) flame spray synthesis [75], (iii) atomic (iv) layer deposition [76] and (v) chemical vapor deposition [77]. AuNPs can be synthesized from molecular components (metal ions) using the above-mentioned methods. All these approaches are suitable for producing small amounts of AuNPs, the shapes and sizes of which depend on the batch used. Sonochemical is a great potential method for AuNP mass production from different materials. This method is a relatively powerful and straightforward technique for producing nanomaterials, and it is possible to control the properties of nanoparticles by modifying the parameters of the ultrasonic process [78,79]. AuNPs with semispherical shapes and average size distributions of about 18.5 were produced through an economical sonochemical method in which the nanoparticles were produced with ultrasounds from droplets of the metal salt precursor solution (Figure 2) [80]. The sonicator probe model (SONICS Ultra-cell MODEL: VC 750) operated for synthesis has an output power, frequency and tip size of 17.9 W·cm^2^, 20 kHz and half an inch, respectively. Under ultrasound waves of 5 min, AuNPs showed high stability in different media (AuNPs in phosphate-buffered saline and water were −39.5 mV and −42.1 mV, respectively). The homogeneity of AuNPs may also be attributed to the intense power and high energy produced by ultrasonic irradiation during the formation and collapse of bubbles, which increase the pressure and temperature of the solution.

**Figure 3:**
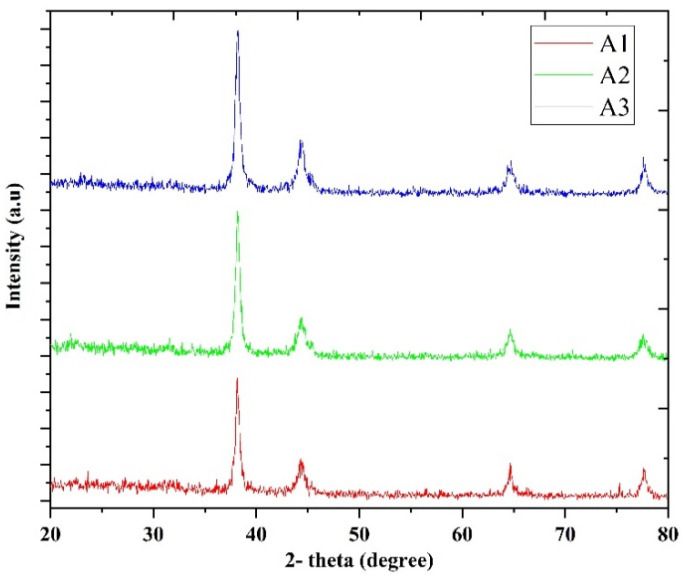
XRD diffractions of AuNPs sample synthesis with different output powers [87]. Copyright 2021 Elsevier.

Yasuda et al. synthesized AuNPs by ultrasonic irradiation at 495 kHz from aqueous solution HAuCl_4_ without reducing the agent or surfactant [81]. The ratio of spherical AuNPs increased with the introduction of ultrafine bubbles (UFBs), and the average diameter of the spherical AuNPs reduced from 119 nm to 22 nm. The reduction of the Au ion was hypothesized to be accelerated by UFBs, because UFBs were nuclei of ultrasonic cavitation, which increased cavitation generation. In addition, AuNP synthesis with UFBs seemed to be stable in the solution, because the AuNPs were immobilized onto UFBs electrostatically, and the lifetimes of UFBs in the liquid were very long. During the pulse-on time, the cavitation and AuNP nucleation improved, whereas, with increasing the pulse-off time, the mean diameter also increased. The AuNPs developed during the pulse-off time. AuNP size regulation in the absence of reducing agents and surfactants was effective by optimizing the number of UFBs and the pulsed ultrasonic conditions. Kumar et al. reported a simple sonochemical method for the preparation of gold–ruthenium (Au–Ru NPs) by co-reduction, and sequential reduction methods were developed [82]. Nanoparticles have the potential for being used in direct methanol fuel cells as catalysts in electrode materials. In addition, the preparation of Au–Ru NPs covered by a polymer was systematically investigated by TEM and a UV-vis spectral study. During the reduction of metal ions, the UV–vis spectral findings suggest that their redox potential governs the order of the reduction process (metal ions), i.e., Au is first reduced, accompanied by ruthenium. Additionally, AuNPs serve as an electron sink to reduce the time needed for Ru^3+^ reduction significantly from 7 h to 4 h. Cui et al. demonstrated that hybrid AuNPs wrapped graphene oxide (GO) materials can be designed and self-assembled through a one-pot sonochemical reaction using HAuCl_4_ and GO precursors [83]. The morphology of the composite materials obtained is that of the AuNP spheres covered with GO sheets like gauze. It is important to use ethylene glycol to synthesize AuNPs, because it is nontoxic. In addition, Au NPs exhibit excellent surface-enhanced Raman scattering in hybrid materials and have also been reported to improve enhanced photocatalytic activity through light irradiation. This method provides a simple way of controlling and tuning the morphology and size of Au NPs in GO-wrapped metal nanoparticles, which expands as a feasible way to produce metal NPs/GO composites employing GO as the precursor material. Wang et al. documented a method of preparing fabrics that can prevent biofilm formation by sonochemical decoration AuNPs covered with N-heterocyclic molecules [84]. Since N-heterocyclic molecules and AuNPs are not toxic to mammalian cells, their research offers a novel strategy to superb antimicrobial activity against multidrug-resistant (MDR) biofilm bacteria in a simple, low-cost and effective manner that holds promise for wide clinical applications. The sonochemical production of AuNPs is recorded utilizing a high-intensity ultrasound (HIU) performing at 463 kHz with various shapes and size distributions [85]. AuNPs are formed by reducing Au^3+^ to Au^0^ by radicals produced through acoustic cavitation. The TEM images revealed that AuNPs exhibit irregular shapes at 30 W, are predominantly icosahedral at 50 W and contain a large amount of 70-W nanorods. AuNP sizes decrease with a narrower size distribution, with increasing acoustic power. The number of radicals formed and the mechanical forces produced control the AuNP size and shape. The TEM images and UV-Vis spectra can be used to indicate a potential explanation for the results observed. The results show that the HIU process can be used to synthesize size- and shape-controlled metal nanoparticles. Radziuk et al. demonstrated that ultrasonic irradiation (20 kHz) for 20 min is enough to fuse the AuNPs in a dumbbell-like structure at the contact [86]. After 60 min of sonication in water, AuNPs acquire a ring-like or worm-like structure. Fused AuNPs with oval or spherical shapes with a size of about 25 nm forms in the presence of dodecyl amine solutions or sodium dodecyl sulfate after ultrasonic irradiation. The dispersion of AuNPs, which is the weakest in pure water, is found as an additional cycle during sonication. The results reported might be of interest to the ultrasonic melting of inorganic materials at the nanoscale to produce metal structures with different properties and morphologies. Recently, by our team, AuNPs with different output powers have been synthesized (A1 = 12, A2 = 20 and A3 = 36 W) [87]. Figure 3 confirms the influence of ultrasounds on the synthesis of various AuNPs as a feature of ultrasound control. The typical diffraction peaks of Au are visible in all samples, but the intensity of the prominent (111) peak for Au is greater at 36 W. This result suggests that the structural properties of the synthesized AuNPs were affected by the output power, as the samples displayed significant differences in peak intensities as the output power was increased. The comparison of the various spectra found that the peak amplitude improved as the ultrasound output power increased. This phenomenon may be due to the distinct characteristics derived from the acoustics.

### 3.3. Fe_3_O_4_@AuNPs

**Figure 4:**
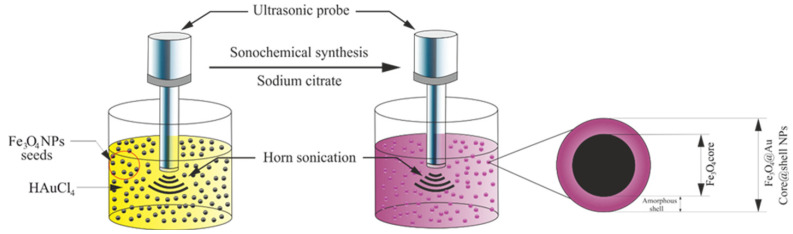
Illustration showing the formation of Fe_3_O_4_@Au NPs using a sonochemical method [32]. Copyright 2019 Elsevier.

Recently, many achievements and scientific outcomes in nanotechnology have contributed to the creation of nanomaterials with the desired structure, chemical and physical compositions [88,89,90,91]. There is currently increasing interest in nanomaterials with multifunctional properties that can be produced for different tasks. Specifically, core–shell nanostructures are increasingly attracting attention because of their universal structures and compositions [92]. The interaction between the shell and core can lead to completely new collective properties in a nanostructure [93,94]. Different organic and inorganic substances can be used as materials for nanostructures’ shells and cores to achieve different effects and physical properties [95]. The differences in the materials used for core–shell nanostructures and, subsequently, their physical characteristics can make use of a new class of nanomaterials with a wide range of applications (e.g., bio-nanotechnology, magnetic devices, nano optics and nanomedicine) [96,97,98,99]. Core–shell nanostructures can be produced by a two-step sequential reaction in which, first, the core is formed and, then, the shell is made up [100]. Fe_3_O_4_@Au (core@shell) is one of the most significant nanoparticles used for many biomedical applications, including catalysis [101], biosensing [102], targeted drug delivery [103], phototherapy [104] and (CT/MRI) dual-modality [105]. Various methodologies were used to synthesize the Fe_3_O_4_ core coated with an Au shell. For instance, direct coating is easy but requires a complicated method of combining two incompatible surfaces. The outcome typically indicates low dispersion and, therefore, fails to synthesize Fe_3_O_4_@Au NPs [106]. In addition, the procedure is time-consuming and laborious [107], as well as produces irregularly shaped and large sizes more than 100 nm, leading to an undesired impact on their medical applications [108]. To successfully synthesize correctly sized and consistently shaped Fe_3_O_4_@Au NPs through a straightforward method remains challenging. A procedure for producing Fe_3_O_4_@Au NPs with outstanding physicochemical characteristics has to be established. The sonochemistry strategy has the ability to develop into an invaluable tool for Au shell deposition on Fe_3_O_4_NPs (Figure 4). The sonochemical procedure influences the surface and structure of Fe_3_O_4_NPs through the acoustic cavity, which inhibits cluster agglomeration and leads to a more stable distribution [32].

**Figure 5:**
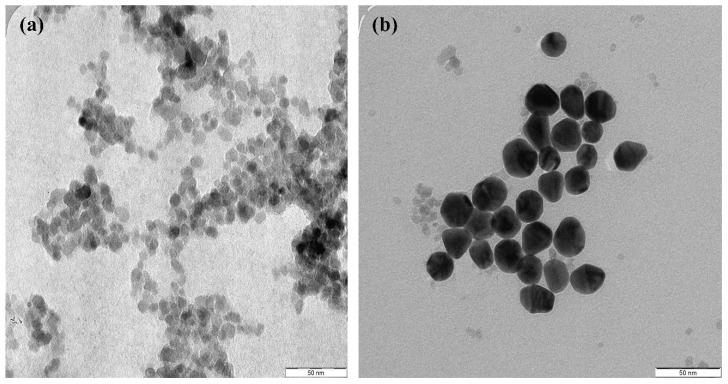
TEM images of (**a**) Fe_3_O_4_NPs and (**b**) Fe_3_O_4_@AuNPs [109]. Copyright 2020 Elsevier.

Fe_3_O_4_@Au NPs have been produced to combine the features of Fe_3_O_4_ NPs and Au NPs with one core@shell nanoparticle [109]. Fe_3_O_4_ NPs were prepared by coprecipitation proceeded by a reduction of the Au shell on the Fe_3_O_4_ surface using a fast and simple sonochemical process within 10 min. Fe_3_O_4_@Au were prepared by employing sodium citrate as a both reducing and capping agent, owing to the inclusion of carboxylate ions and then identified by several methods that verified the development of the Au shell on the Fe_3_O_4_ surface. Figure 5 provides TEM images of Fe_3_O_4_NPs and Fe_3_O_4_ after being coated by AuNPs. SPR peaks shifted from 521 nm to 541 nm, indicating that the Au NPs shell is tightly adsorbed to the Fe_3_O_4_ surface. Sonochemically produced Fe_3_O_4_@Au NPs have high magnetization saturation values even after being coated with an Au shell. The high energy generated by ultrasonic irradiation enhances the covalent interaction between the core of Fe_3_O_4_ and the Au shell, even though the synthesis period was especially short. In addition, the obtained Fe_3_O_4_@Au NPs showed good biocompatibility and high transverse relaxation, as well as X-ray attenuation values, which were higher than those recorded for NPs provided by conventional approaches and commercial NPs.

**Figure 6:**
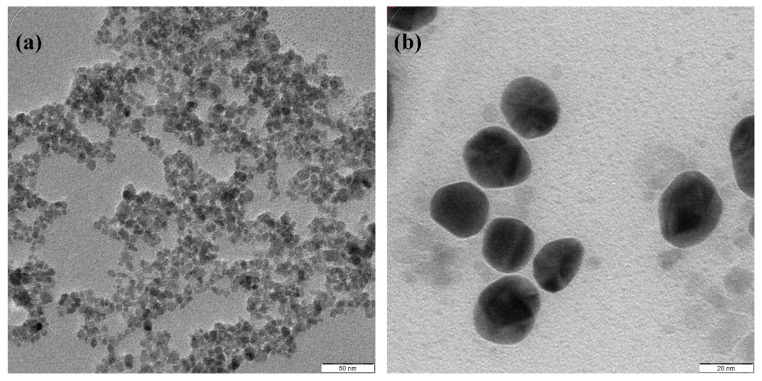
TEM images of (**a**) Fe_3_O_4_ and (**b**) Fe_3_O_4_@Au NPs [110]. Copyright 2020 Elsevier.

**Figure 7:**
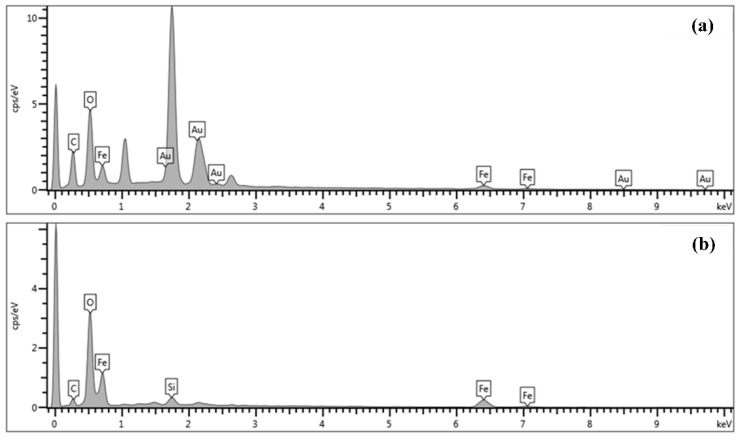
Energy-dispersive X-ray (EDX) of (**a**) Fe_3_O_4_@Au NPs and (**b**) Fe_3_O_4_ [110]. Copyright 2020 Elsevier.

Spherical Fe_3_O_4_@AuNPs were successfully produced as a photothermal agent with a mean size of 20.8 nm by a sonochemical process [104]. At 40 kHz (ultrasound frequency), 5-mg Fe_3_O_4_NPs were ultrasonically dispersed for 15 min in 20 mL of sodium citrate. Subsequently, a freshly formulated HAuCl_4_ (10 mL, 0.1 M) solution was applied in order to minimize HAuCl_4_ and shape the shell based on Fe_3_O_4_. The process of sonication proceeded for 15 min. A permanent magnet was used for collecting Fe_3_O_4_@AuNPs and washed thoroughly with distilled water and re-dissipated in distilled water. The Fe_3_O_4_@AuNPs cell viability assessment showed that MCF-7 lines have negligible toxicity, even with high levels of NPs, after 24 h. After illumination with a laser at 808 nm (200 mW, 10 min), MCF-7 cells treated with Fe_3_O_4_@ Au NPs were significantly reduced (73.9%) at 50-µgFe/mL viability. In this paper, results agree that the produced Fe_3_O_4_@AuNPs that pose a threat to human health could be used to increase breast cancer treatment as a photothermal therapist. The synthesis of Fe_3_O_4_@AuNPs and variations of the parameters were also further optimized using the surface response method (RSM) approach [110]. Experimental sequences of 14 different variations in sonication amplitude, sodium citrate and HAuCl_4_ concentrations were performed using RSM to optimize the experimental conditions, and a variance analysis (ANOVA) was used to achieve the optimum parameters for the experimental results. The target of the zeta potential of −46.125 mV was obtained with an optimum sonication amplitude of 40%, 30 mL of sodium citrate and 10 mL of HAuCl_4_, which was consistent (about 99.2%) with the actual average of zeta potential (−45.8 mV). The results confirmed that the sonochemical method effectively synthesized monodispersed and highly stable Fe_3_O_4_@Au NPs with an average size diameter of 20 nm in less than 8 min. Figure 6 shows the TEM images of Fe_3_O_4_ and Fe_3_O_4_@Au NPs where the initial Fe_3_O_4_ is a homogeneous and spherical-shaped with an average size of about 8.7 nm. The resultant Fe_3_O_4_@Au NPs remained spherically shaped but increased in the size diameter around 20 nm, caused by the existence of the Au shell covering of the Fe_3_O_4_ core (Figure 6b). Figure 7 illustrates the EDX analysis of Fe_3_O_4_ and Fe_3_O_4_@Au NPs. The elements Fe, Au, C and O were uniformly distributed in the sample, supporting the coating of the Au shell on the surface of Fe_3_O_4_NPs. The presence of uncoated iron, as confirmed by TEM, is seen in Figure 7b. The Au shell thickness was around 11 nm and could be changed by various experimental conditions [111].

Another sonochemical method for developing Fe_3_O_4_@Au NPs was implemented by synthesizing Au layers on the Fe_3_O_4_ surface, with sodium citrate being used as a reductant [112]. The TEM images showed that both Fe_3_O_4_ and Fe_3_O_4_@Au NPs are highly uniformly spherical [113]. The mean diameter ranges were approximately 9 nm and 27 nm for Fe_3_O_4_NPs and Fe_3_O_4_@Au NPs, respectively. The Au shell was about 9 nm thick. The thickness of the shell can be modified by adjusting the experimental parameters [114]. Hu et al. reported that a simple but effective method (ultrasonic process (40 kHz, 50 W)) was used to produce Au-coated Fe_3_O_4_ with polyphosphazene (PZS) as a mediator [115]. PZS is an appropriate layer of glue, because it has phenolic hydroxyl groups. Fe_3_O_4_NPs were prepared under high temperatures from the iron salt precursor compound with polyol medium triethylene-glycol using ultrasonic power to irradiate a combination of hexachlorocyclotriphosphazene, triethylamine and Fe_3_O_4_. Thereafter, PZS was added to the mixture, then irradiated about 6 h; any adverse effect of long-time irradiation was not mentioned by the authors. The Fe_3_O_4_NPs-PZS were then mixed in a HAuCl_4_ solution under room temperature. After 30 min of ultrasonic waves, sodium citrate was added to the mixture. As a result, the Fe_3_O_4_-PZS-Au NPs could become an excellent candidate for photothermal therapy, as well as other applications, due to the characteristics of the Au shell.

Aziz et al. showed a simple procedure to produce Fe_3_O_4_@Au NPs using ultrasonic power that requires a chemical reduction in the existence of APTES ((3-aminopropyl) triethoxysilane)-coated Fe_3_O_4_NPs seeded through chilled sodium borohydride [116]. The acoustic cavitation effect provides high pressure and high thermal energy. On the Fe_3_O_4_ surface, the Au NPs establish a covalent connection to the terminal amine. The creation of Au-coated Fe_3_O_4_NPs is stated by a change in the color of the combination to dark purple [107,117]. The observable purple color can be related to the purple or red-to-blue change in Au NP surface plasmon resonance (SPR), which is attributable in turn to Au NP conjugation with Fe_3_O_4_. Furthermore, Fe_3_O_4_@Au NPs of quite high saturation magnetization were synthesized with an easy and rapid sonochemical process (60 kHz). In the following sequence, the synthesis involved three-phase reactions: (i) producing Fe_3_O_4_NPs via the co-precipitation technique (ii), coated Fe_3_O_4_ by the amine group and (iii) Au^3+^ ion reductions with the assistance of sonication waves [37]. Chemically, the ultrasound influences the activity of the nanoparticle surface through acoustic cavitation. Unlike the regular stirring method, the ultrasound method is valuable in obtaining a uniform shape, removing the variables of localized conditions, speeding up the reaction rate and developing a new phase. This procedure also reveals a shearing effect for agglomeration, which is important for high monodispersity nanoparticle synthesis. Fe_3_O_4_@Au NPs were synthesized ultrasonically with an average diameter about 9–25 nm as a stationary phase with high dispersibility, magnetic responsivity and excellent water solubility. After that, bovine serum albumin (BSA) was grafted on the Fe_3_O_4_@Au NPs surface by conjugating Au NPs with BSA to produce Fe_3_O_4_@Au NPs-BSA [118]. Fe_3_O_4_@Au-BSA have a high magnetization that enables them to be activated easily through an external magnetic field, as well as a large area and excellent biocompatibility of the Au shell. Fe_3_O_4_@Au NPs-BSA has developed a new approach in addition to novel applications of magnetic NPs in studies of large concentrations of protein target enantiomers. All in all, the results confirm that the use of sonochemical for the production of metal nanostructures provides highly stable, eco-friendly and cost-effective particularly nontoxic nanoparticles with good morphology and high-quality crystal structures [80].

**Table 1:** Summary of the recently published studies on Fe_3_O_4_, AuNPs and Fe_3_O_4_@AuNPs syntheses using sonochemicals.

No.	Nanoparticles	Power or Frequency	Size and Shape	Media	Precursor Concentration	SPR	Magnetization	Ref.
1	Fe_3_O_4_	130 kHz	180 nm	NH_4_Cl	0.5 mol/L	……….	20 mT	[50]
2	Fe_3_O_4_@GOS	60 W	96 nm spherical	sodium acetate Polyvinylpyrrolidone	2 mg	……….	……….	[51]
3	Fe_3_O_4_	……….	15 nm amorphous	NH_4_OH	100 mg/L	215 nm	76.89 emu/g	[52]
4	Fe_3_O_4_@MWCNTs	40 kHz	20 nm amorphous	water/ethylene glycol	40 mg	……….	……….	[53]
5	Fe_3_O_4_	40 kHz, 150 W	22.41 nm Semi-spherical	ethylenediamine	……….	……….	54.24	[54]
6	Fe_3_O_4_	20 kHz 1500 W	80 nm cubes	distilled water	2.31 mg		91 emu/g	[55]
7	AuNPs	20 kHz/17.9 W·cm^2^	18 nm semi-spherical	distilled water	0.03 M	520 nm	……….	[80]
8	AuNPs	495 kHz	22 nm spherical	aqueous solution	0.1 mM	530 nm	……….	[81]
9	Au–Ru NPs	355 kHz	15 nm spherical	polyethylene glycol perchloric acid	5 × 10^−5^ M	536 nm	……….	[82]
10	GO-wrapped Au NPs	200 W	500 nm sphere	water and ethylene glycol	(8 mg, 0.02 mmol)	546 nm	……….	[83]
11	N_Au NPs	750 W	4.83 nm spherical	methanol	0.1 mmol	……….	……….	[84]
12	AuNPs	463 kHz	14 nm nanorods	water	0.2 mM	650 nm	……….	[85]
13	AuNPs	20 kHz 40 W.cm^−2^	25 nm oval or spherical	dodecyl amine solutions or sodium dodecyl sulfate	1 mmol/L	523 nm	……….	[86]
14	Fe_3_O_4_@AuNPs	20 kHz/750 W	21 nm spherical	water	0.03 M	541 nm	42.2 emu/g	[109]
15	Fe_3_O_4_@AuNPs	40 kHz	20.8 nm spherical	water	0.1 M	538 nm	……….	[104]
16	Fe_3_O_4_@AuNPs	20 kHz and 750 w	20 nm spherical	water	0.3 M	545 nm	54 emu/g	[110]
17	Fe_3_O_4_-PZS-Au NPs	40 kHz, 50 W	253 nm spherical	NaBH4	24 mM	526 nm	24.2 emu/g	[115]
18	Fe_3_O_4_@Au NPs-BSA	60 kHz	9–25 nm spherical	sodium citrate	15 mL (1% mass fraction)	547 nm	……….	[118]

## 4. Shapes of Synthesis Nanomaterials Sonochemically

**Figure 8:**
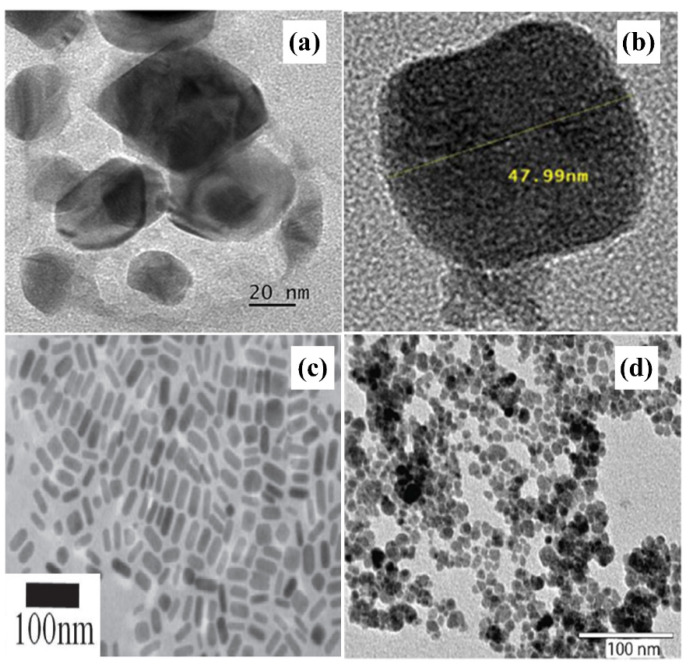
Sonochemical method synthesis different shapes. (**a**) TEM image of the gold nanodisc [119]. Copyright 2013 Elsevier. (**b**) Single-particle Fe_3_O_4_NPs [121]. Copyright 2018 Elsevier. (**c**) TEM images of gold nanorods fabricated at 1 min [122] Copyright 2014 Elsevier., and (**d**) TEM image of a Fe_3_O_4_ nano-sphere [123]. Copyright 2017 American Chemical Society.

Study groups around the world have, over the years, obtained nanoproducts with unusual shapes, such as nanorods, nanotubes, sphere, non-sphere, nano-hexagonal and others. This section will clarify the capacity of the sonochemical method to generate a variety of shapes for nanoparticles. Wani and Ahmad produced polyhedral structures and nanodiscs of AuNPs through the sonochemical technique without any stabilizer (Figure 8a) [119]. Sodium borohydride was used as a reducing agent and has the potential to produce a mixture of nanocrystals of various morphologies, including hexagons, cubes and other polyhedral forms with an average size of 30 nm (Table 2). Moreover, Jung et al. developed nano-cubic C-Fe_2_O_3_@Au sonochemically [120]. The Fe_2_O_3_@Au cubic is formed by applying ultrasound waves to both C- Fe_2_O_3_ and HAuCl_4_. Au-coated C- Fe_2_O_3_ was produced under ultrasonic irradiation (300 W, 20 kHz) for 30 min without any supporting agents. The precipitate was obtained at 8000 rpm by centrifugation and washed with distilled water three times to remove the residual impurities. The sonochemistry method provided a direct synthesis technique for the hematite cubic form decorated with nano-sized Au, suggesting possible applications for lithium storage materials, catalytic support and water-splitting. A new type of nanocube with a size of about 43 nm was produced sonochemically in 30 min (Figure 8b) [121]. The sonochemical formation of Fe_3_O_4_NPs does not require strict experimental procedures or any toxic agent, and therefore, it is a fast, green, efficient and straightforward method to produce extremely active catalysts for the treatment of environmental pollutants.

The mechanism by which sonochemically the nanorods are formed is much simpler. Okitsu et al. reported a rapid sonochemical route (one-pot synthesis) for the production gold nanorods in the aqueous solution in the existence of ascorbic acid, silver nitrate and cetyltrimethylammonium bromide in a short time [122]. The role of time, concentration, sonication time and capping agent as effective variables has been investigated for the formation of gold nanorods via the sonochemical route. TEM image shows the histogram of the rods diameter of gold was about of 34 nm (Figure 8c). In growth investigations of gold seeds formed by sonochemistry, the induction time before growth began has been longer for shorter periods of irradiation. The result showed that the number of gold seeds that were formed by sonochemistry increased with an increase in irradiation time. The gold nanorods provided were wider and longer when the time of irradiation time was shorter. Nagvenkar et al. and Mohammadi et al. have also demonstrated the potential for fabrication nanorods in inorganic nanoparticles using the sonochemistry method [126,127].

In the case of spherical shape, Davino et al. employed the sonochemical method to prepare 9–11 nm spheres Fe_3_O_4_NPs with excellent physicochemical properties in 12 min (Figure 8d) [123]. A two-step synthesis with an ultrasound probe with a frequency of (585 W, 20 kHz) was used to synthesize and conjugate Fe_3_O_4_NPs. In addition, even with the overlay with a non-magnetic material, the sonochemistry approach generated Fe_3_O_4_NPs with high magnetization values about 77 emu/g. The high energy generated by ultrasound waves allowed the covalent bonding between the Fe_3_O_4_ and capping agent molecules, given the relatively short time used during the synthesis. Fe_3_O_4_NPs coated with amine and carboxylate displayed strong colloidal stability in water which makes them promise for in vivo applications. In particular, the procedure used here overcomes the current synthesizing-related limitations of biomedical research on Fe_3_O_4_NPs from the bench to the clinics. It offers a simple experimental treatment that may bring new opportunities for the scale-up of structured Fe_3_O_4_NPs with outstanding physicochemical features suitable for biomedical usage.

In a similar study, Nazrul Islam et al. reported a simple sonochemical procedure was introduced to synthesize the spherical Fe_3_O_4_NPs, which were effectively synthesized using cheap and non-toxic metal salts as reaction mixture [124]. It is also worth noting that TEM measurements revealed that the as Fe_3_O_4_NPs were produced to have a size distribution (11 nm) in a small range and progressive monodispersity. The measurement curve for magnetization indicates that Fe_3_O_4_NPs have superparamagnetic behavior (80 emu/g), which is very similar to the bulk value of Fe_3_O_4_. They mentioned that this approach can provide an effective and quick synthetic route for bio synthesizing Fe_3_O_4_NPs and many other applications as well. In a different study, Bagheri et al. stated that a facile sonochemical method was proposed to optimize spherical Fe_3_O_4_@AuNPs [128]. The optimum values of concentration, pH, sonication time and adsorbent weight were 26.5 mg L^−1^, 4.0, 4 min, 0.25, respectively. The ANOVA revealed a strong determination coefficient (Adj-R^2^ > 0.920, and R^2^ > 0.972). The rapid and rapid transfer of dyes to the adsorbent surface allows for rapid balance which confirms the suitability and efficiency of ultrasonic power as a powerful wastewater treatment tool. The results indicate that the initial adsorption is very fast due to the high active surface area and an adsorbent vacant site that enhances the interface and a driving force. In addition, the results of this study encourage research and industry to use ultrasound devices for more efficient synthesizing nanomaterials. Takahashi et al. demonstrated an easy and effective post-modification pathway was developed to modify the surface charge of AuNPs using surfactant-free (thioctic acid) AuNPs produced via the sonochemical method [125]. The AuNPs have been synthesized in 30 min by a sonochemical surfactant-free reaction with ultrasound at 430 kHz. SEM images verified that nano spherical of AuNPs (15–20 nm) was formed during the sonication of a 200-μM HAuCl_4_ aqueous solution. It has been found that the presence of trace nereistoxin might cause AuNPs aggregation if thioctic acid was partially covered at pH 5 on the AuNPs surface. Since the aggregation could not have been produced without thiol groups by the other amine compounds, it showed that the pronounced nereistoxin concentration might reduce the thioctic acid surface charge covered AuNPs resulting in aggregation. A simplistic liquid-liquid reverse extraction method has been successfully used to prove the ability of the proposed method for detecting trace levels of nereistoxin in environmental water samples.

## 5. Conclusions

Sonochemistry demonstrates the use of modern methods and techniques and has proven to be more simple and rapid in some ways than mature and conventional ones for synthesizing Fe_3_O_4_, Au and Fe_3_O_4_@Au NPs. With simple alterations of the precursor compositions and reaction conditions, the usage of a high-intensity ultrasound has effectively prepared a multitude of nanostructured materials for controlled structures, morphologies and compositions. Using such exceptional conditions, naturally presented by acoustic bubbles, a large variety of nanomaterials were sonochemically synthesized even without the assistance of extensive and costly equipment or facilities. Sonochemistry helps to eliminate the complexity and to improve the handling of materials. The sonochemical method has demonstrated its ability to produce diverse desired sizes and shapes of Fe_3_O_4_, Au and Fe_3_O_4_@Au NPs. This review successfully highlighted its significant contributions and progress, in addition to precise discussions on the sonochemical synthesis of nanomaterials.

## Figures and Tables

**Table 2:** Summary of the synthesis of various shapes using sonochemistry.

No	Particles Shape	Particles Size (nm)	Ultrasonic Power	Ref
1	nanodiscs	30	……….	[119]
2	nanocubic	433	(300 W, 20 kHz)	[120]
3	nanocube	43	……….	[121]
4	nanorods	34		[122]
5	nanosphere	9–11	(585 W, 20 kHz)	[123]
6	nanosphere	11	……….	[124]
7	nanosphere	15–20	430 kHz	[125]

## Data Availability

Not applicable.

## References

[1] Tsakalakos T., Ovid’ko I.A., Vasudevan A.K. (2012). Nanostructures: Synthesis, Functional Properties and Application.

[2] Jameel M.S., Aziz A.A., Dheyab M.A. (2021). Impacts of various solvents in ultrasonic irradiation and green synthesis of platinum nanoparticle. Inorg. Chem. Commun..

[3] Jameel M.S., Aziz A.A., Dheyab M.A. (2020). Comparative analysis of platinum nanoparticles synthesized using sonochemical-assisted and conventional green methods. Nano Struct. Nano Objects.

[4] Richards W.T., Loomis A.L. (1927). The Chemical Effects of High Frequency Sound Waves I. A preliminary survey. J. Am. Chem. Soc..

[5] Okoli C.U., Kuttiyiel K.A., Cole J., McCutchen J., Tawfik H., Adzic R.R., Mahajan D. (2018). Solvent effect in sonochemical synthesis of metal-alloy nanoparticles for use as electrocatalysts. Ultrason. Sonochem..

[6] Aufaure R., Lalatonne Y., Lièvre N., Heintz O., Motte L., Guénin E. (2014). One pot microwave assisted synthesis of bisphosphonate alkene capped gold nanoparticles. RSC Adv..

[7] Sangnier A.P., Aufaure R., Cheong S., Motte L., Palpant B., Tilley R.D., Guenin E., Wilhelm C., Lalatonne Y. (2019). Raspberry-like small multicore gold nanostructures for efficient photothermal conversion in the first and second near-infrared windows. Chem. Commun..

[8] Hu H., Yang H., Huang P., Cui D., Peng Y., Zhang J., Lu F., Lian J., Shi D. (2010). Unique role of ionic liquid in microwave-assisted synthesis of monodisperse magnetite nanoparticles. Chem. Commun..

[9] Kiruba V.S.A., Dakshinamurthy A., Selvakumar P.M. (2013). Eco-Friendly Biocidal Silver-Activated Charcoal Nanocomposite: Antimicrobial Application in Water Purification. Synth. React. Inorg. Met. Chem..

[10] Oyetade O., Oyeleke G., Adegoke B., Akintunde A. (2012). Stability studies on ascorbic acid (vitamin C) from different sources. J. Appl. Chem..

[11] Hinman J.J., Suslick K.S. (2017). Nanostructured Materials Synthesis Using Ultrasound. Sonochemistry.

[12] Li Z., Zhuang T., Dong J., Wang L., Xia J., Wang H., Cui X., Wang Z. (2021). Sonochemical fabrication of inorganic nanoparticles for applications in catalysis. Ultrason. Sonochem..

[13] Li Z., Dong J., Zhang H., Zhang Y., Wang H., Cui X., Wang Z. (2021). Sonochemical catalysis as a unique strategy for the fabrication of nano-/micro-structured inorganics. Nanoscale Adv..

[14] Xu H., Zeiger B.W., Suslick K.S. (2013). Sonochemical synthesis of nanomaterials. Chem. Soc. Rev..

[15] Suslick K.S., Flannigan D.J. (2008). Inside a Collapsing Bubble: Sonoluminescence and the Conditions During Cavitation. Annu. Rev. Phys. Chem..

[16] Dheyab M.A., Aziz A.A., Jameel M.S., Abu Noqta O., Mehrdel B. (2020). Synthesis and coating methods of biocompatible iron oxide/gold nanoparticle and nanocomposite for biomedical applications. Chin. J. Phys..

[17] Bang J.H., Suslick K.S. (2010). Applications of Ultrasound to the Synthesis of Nanostructured Materials. Adv. Mater..

[18] Dheyab M.A., Aziz A.A., Khaniabadi P.M., Jameel M.S., Ahmed N.M., Ali A.T. (2020). Distinct advantages of using sonochemical over laser ablation methods for a rapid-high quality gold nanoparticles production. Mater. Res. Express.

[19] Rahimi M., Movahedirad S., Shahhosseini S. (2017). CFD study of the flow pattern in an ultrasonic horn reactor: Introducing a realistic vibrating boundary condition. Ultrason. Sonochem..

[20] Vabbina P.K., Karabiyik M., Al-Amin C., Pala N., Das S., Choi W., Saxena T., Shur M. (2013). Controlled Synthesis of Single-Crystalline ZnO Nanoflakes on Arbitrary Substrates at Ambient Conditions. Part. Part. Syst. Charact..

[21] Kristl M., Ban I., Danč A., Danč V., Drofenik M. (2010). A sonochemical method for the preparation of cadmium sulfide and cadmium selenide nanoparticles in aqueous solutions. Ultrason. Sonochem..

[22] García D.A., Mendoza L., Vizuete K., Debut A., Arias M.T., Gavilanes A., Terencio T., Ávila E., Jeffryes C., Dahoumane S.A. (2020). Sugar-Mediated Green Synthesis of Silver Selenide Semiconductor Nanocrystals under Ultrasound Irradiation. Molecules.

[23] Kumar B., Smita K., Cumbal L., Debut A., Pathak R.N. (2014). Sonochemical Synthesis of Silver Nanoparticles Using Starch: A Comparison. Bioinorg. Chem. Appl..

[24] Mizukoshi Y., Oshima R., Maeda Y., Nagata Y. (1999). Preparation of Platinum Nanoparticles by Sonochemical Reduction of the Pt(II) Ion. Langmuir.

[25] Noman M.T., Petru M., Militký J., Azeem M., Ashraf M.A. (2019). One-Pot Sonochemical Synthesis of ZnO Nanoparticles for Photocatalytic Applications, Modelling and Optimization. Materials.

[26] Taghavinezhad P., Haghighi M., Alizadeh R. (2018). Sonosynthesis of VOx/MCM-41 nanocatalyst enhanced by various metal oxides (Mg, AL, Zr) for CO2-oxidative dehydrogenation of ethane to ethylene. Microporous Mesoporous Mater..

[27] Bedini A.P.C., Klingebiel B., Luysberg M., Carius R. (2017). Sonochemical synthesis of hydrogenated amorphous silicon nanoparticles from liquid trisilane at ambient temperature and pressure. Ultrason. Sonochem..

[28] Shi L., Zhang G., Wang Y. (2018). Tailoring catalytic performance of carbon nanotubes confined CuOCeO_2_ catalysts for CO preferential oxidation. Int. J. Hydrogen Energy.

[29] Lin J.-H., Tsao Y.-H., Wu M.-H., Chou T.-M., Lin Z.-H., Wu J.M. (2017). Single-and few-layers MoS_2_ nanocomposite as piezo-catalyst in dark and self-powered active sensor. Nano Energy.

[30] Sostaric J.Z., Mulvaney P., Grieser F. (1995). Sonochemical dissolution of MnO_2_ colloids. J. Chem. Soc. Faraday Trans..

[31] Makino K., Mossoba M.M., Riesz P. (1982). Chemical effects of ultrasound on aqueous solutions. Evidence for hydroxyl and hydrogen free radicals (.cntdot.OH and.cntdot.H) by spin trapping. J. Am. Chem. Soc..

[32] Dheyab M.A., Aziz A.A., Jameel M.S., Khaniabadi P.M., Mehrdel B. (2019). Mechanisms of effective gold shell on Fe_3_O_4_ core nanoparticles formation using sonochemistry method. Ultrason. Sonochem..

[33] Suslick K.S., Price G.J. (1999). Applications of Ultrasound to Materials Chemistry. Annu. Rev. Mater. Sci..

[34] Yoon Y.-J., Kang S.-H., Do C., Moon S.Y., Kim T.-H. (2019). Water-Redispersible and Highly Stable Gold Nanoparticles Permanently Capped by Charge-Controllable Surfactants for Potential Medical Applications. ACS Appl. Nano Mater..

[35] Karimzadeh I., Aghazadeh M., Dalvand A., Doroudi T., Kolivand P.H., Ganjali M.R., Norouzi P. (2017). Effective electrosynthesis and in situ surface coating of Fe 3 O 4 nanoparticles with polyvinyl alcohol for biomedical applications. Mater. Res. Innov..

[36] Izadiyan Z., Shameli K., Miyake M., Teow S.-Y., Peh S.-C., Mohamad S.E., Taib S.H.M. (2019). Green fabrication of biologically active magnetic core-shell Fe_3_O_4_/Au nanoparticles and their potential anticancer effect. Mater. Sci. Eng. C.

[37] Wu W., He Q., Chen H., Tang J., Nie L. (2007). Sonochemical synthesis, structure and magnetic properties of air-stable Fe_3_O_4_/Au nanoparticles. Nanotechnology.

[38] Ashokkumar M. (2011). The characterization of acoustic cavitation bubbles—An overview. Ultrason. Sonochem..

[39] Suslick K.S., Didenko Y., Fang M.M., Hyeon T., Kolbeck K.J., McNamara W.B., Mdleleni M.M., Wong M. (1999). Acoustic cavitation and its chemical consequences. Philos. Trans. R. Soc. A Math. Phys. Eng. Sci..

[40] Wolloch L., Kost J. (2010). The importance of microjet vs shock wave formation in sonophoresis. J. Control. Release.

[41] Shekhar A., Nomura K.-I., Kalia R.K., Nakano A., Vashishta P. (2013). Nanobubble Collapse on a Silica Surface in Water: Billion-Atom Reactive Molecular Dynamics Simulations. Phys. Rev. Lett..

[42] Doktycz S.J., Suslick K.S. (1990). Interparticle collisions driven by ultrasound. Science.

[43] Putterman S. (1995). Sonoluminescence: Sound into light. Sci. Am..

[44] Suslick K.S. (1994). The Chemistry of Ultrasound.

[45] Laurent S., Forge D., Port M., Roch A., Robic C., Elst L.V., Muller R.N. (2008). Magnetic Iron Oxide Nanoparticles: Synthesis, Stabilization, Vectorization, Physicochemical Characterizations, and Biological Applications. Chem. Rev..

[46] Dang F., Enomoto N., Hojo J., Enpuku K. (2009). Sonochemical synthesis of monodispersed magnetite nanoparticles by using an ethanol–water mixed solvent. Ultrason. Sonochem..

[47] Marchegiani G., Imperatori P., Mari A., Pilloni L., Chiolerio A., Allia P.M.E.I., Tiberto P., Suber L. (2012). Sonochemical synthesis of versatile hydrophilic magnetite nanoparticles. Ultrason. Sonochem..

[48] Shafi K.V., Ulman A., Yan X., Yang N.L., Estournes C., White H., Rafailovich M. (2001). Sonochemical Synthesis of Functionalized Amorphous Iron Oxide Nanoparticles. Langmuir.

[49] Mukh-Qasem R.A., Gedanken A. (2005). Sonochemical synthesis of stable hydrosol of Fe_3_O_4_ nanoparticles. J. Colloid Interface Sci..

[50] Lüdtke-Buzug K., Penxová Z. (2019). Superparamagnetic Iron Oxide Nanoparticles: An Evaluation of the Sonochemical Synthesis Process. Curr. Dir. Biomed. Eng..

[51] Sriram B., Govindasamy M., Wanga S.-F., Ramalingam R.J., Al-Lohedanb H., Maiyalagan T. (2019). Novel sonochemical synthesis of Fe_3_O_4_ nanospheres decorated on highly active reduced graphene oxide nanosheets for sensitive detection of uric acid in biological samples. Ultrason. Sonochem..

[52] Villegas V.A.R., Ramírez J.I.D.L., Guevara E.H., Sicairos S.P., Ayala L.A.H., Sanchez B.L. (2020). Synthesis and characterization of magnetite nanoparticles for photocatalysis of nitrobenzene. J. Saudi Chem. Soc..

[53] Wu X., Xu G., Zhu J.-J. (2019). Sonochemical synthesis of Fe_3_O_4_/carbon nanotubes using low frequency ultrasonic devices and their performance for heterogeneous sono-persulfate process on inactivation of Microcystis aeruginosa. Ultrason. Sonochem..

[54] Boustani K., Shokri A., Shayesteh S.F., Jafari A. (2020). Ultrasound-Assisted Synthesis and Tuning the Magnetic and Structural Features of Superparamagnetic Fe_3_O_4_ Nanoparticles by Using Ethylenediamine as a Precipitating Agent. J. Supercond. Nov. Magn..

[55] Abbas M., Takahashi M., Kim C. (2012). Facile sonochemical synthesis of high-moment magnetite (Fe_3_O_4_) nanocube. J. Nanoparticle Res..

[56] Penders J., Stolzoff M., Hickey D.J., Andersson M., Webster T.J. (2017). Shape-dependent antibacterial effects of non-cytotoxic gold nanoparticles. Int. J. Nanomed..

[57] Owaid M.N., Rabeea M.A., Aziz A.A., Jameel M.S., Dheyab M.A. (2019). Mushroom-assisted synthesis of triangle gold nanoparticles using the aqueous extract of fresh Lentinula edodes (shiitake), Omphalotaceae. Environ. Nanotechnol. Monit. Manag..

[58] Dheyab M.A., Owaid M.N., Rabeea M.A., Aziz A.A., Jameel M.S. (2020). Mycosynthesis of gold nanoparticles by the Portabello mushroom extract, Agaricaceae, and their efficacy for decolorization of Azo dye. Environ. Nanotechnol. Monit. Manag..

[59] Huang H., Du Toit H., Ben Jaber S., Wu G., Panariello L., Thanh N.T.K., Parkin I.P., Gavriilidis A. (2019). Rapid synthesis of gold nanoparticles with carbon monoxide in a microfluidic segmented flow system. React. Chem. Eng..

[60] Golub D., Ivanič A., Majerič P., Tiyyagura H.R., Anžel I., Rudolf R. (2019). Synthesis of Colloidal Au Nanoparticles through Ultrasonic Spray Pyrolysis and Their Use in the Preparation of Polyacrylate-AuNPs’ Composites. Materials.

[61] Adekoya J.A., Ogunniran K.O., Siyanbola T.O., Dare E.O., Revaprasadu N., Seehra M., Bristow A.D. (2018). Band Structure, Morphology, Functionality, and Size-Dependent Properties of metal Nanoparticles. Noble and Precious Metals—Properties, Nanoscale Effects and Applications.

[62] Mehrdel B., Othman N., Aziz A.A., Khaniabadi P.M., Jameel M.S., Dheyab M.A., Amiri I.S. (2019). Identifying Metal Nanoparticle Size Effect on Sensing Common Human Plasma Protein by Counting the Sensitivity of Optical Absorption Spectra Damping. Plasmonics.

[63] Majidi S., Sehrig F.Z., Farkhani S.M., Goloujeh M.S., Akbarzadeh A. (2014). Current methods for synthesis of magnetic nanoparticles. Artif. Cells Nanomed. Biotechnol..

[64] Yu X., Jiao Y., Chai Q. (2016). Applications of Gold Nanoparticles in Biosensors. Nano Life.

[65] Tiwari P.M., Vig K., Dennis V.A., Singh S.R. (2011). Functionalized Gold Nanoparticles and Their Biomedical Applications. Nanomaterials.

[66] Chen J., Ning C., Zhou Z., Yu P., Zhu Y., Tan G., Mao C. (2019). Nanomaterials as photothermal therapeutic agents. Prog. Mater. Sci..

[67] Song W., Ge S. (2019). Application of Antimicrobial Nanoparticles in Dentistry. Molecules.

[68] Balhaddad A.A., Kansara A.A., Hidan D., Weir M.D., Xu H.H., Melo M.A.S. (2019). Toward dental caries: Exploring nanoparticle-based platforms and calcium phosphate compounds for dental restorative materials. Bioact. Mater..

[69] Suchomel P., Kvitek L., Prucek R., Panacek A., Halder A., Vajda S., Zboril R. (2018). Simple size-controlled synthesis of Au nanoparticles and their size-dependent catalytic activity. Sci. Rep..

[70] Mieszawska A.J., Mulder W.J.M., Fayad Z.A., Cormode D.P. (2013). Multifunctional Gold Nanoparticles for Diagnosis and Therapy of Disease. Mol. Pharm..

[71] Khan A., Rashid R., Murtaza G., Zahra A. (2014). Gold Nanoparticles: Synthesis and Applications in Drug Delivery. Trop. J. Pharm. Res..

[72] Majerič P., Jenko D., Friedrich B., Rudolf R. (2017). Formation mechanisms for gold nanoparticles in a redesigned Ultrasonic Spray Pyrolysis. Adv. Powder Technol..

[73] Rabeea M.A., Owaid M.N., Aziz A.A., Jameel M.S., Dheyab M.A. (2020). Mycosynthesis of gold nanoparticles using the extract of Flammulina velutipes, Physalacriaceae, and their efficacy for decolorization of methylene blue. J. Environ. Chem. Eng..

[74] Shariq M., Friedrich B., Budic B., Hodnik N., Ruiz-Zepeda F., Majerič P., Rudolf R. (2018). Successful synthesis of gold nanoparticles through ultrasonic spray pyrolysis from a gold (III) nitrate precursor and Their interaction with a high electron beam. ChemistryOpen.

[75] Teoh W.Y. (2013). A Perspective on the Flame Spray Synthesis of Photocatalyst Nanoparticles. Materials.

[76] Griffiths M.B.E., Pallister P.J., Mandia D.J., Barry S.T. (2015). Atomic Layer Deposition of Gold Metal. Chem. Mater..

[77] Palgrave R.G., Parkin I.P. (2007). Aerosol Assisted Chemical Vapor Deposition of Gold and Nanocomposite Thin Films from Hydrogen Tetrachloroaurate (III). Chem. Mater..

[78] Fu J., Daanen N.N., Rugen E.E., Chen D.P., Skrabalak S.E. (2017). Simple Reactor for Ultrasonic Spray Synthesis of Nanostructured Materials. Chem. Mater..

[79] Hu X., Takada N., Machmudah S., Wahyudiono, Kanda H., Goto M. (2020). Ultrasonic-Enhanced Fabrication of Metal Nanoparticles by Laser Ablation in Liquid. Ind. Eng. Chem. Res..

[80] Dheyab M.A., Aziz A.A., Jameel M.S., Khaniabadi P.M., Oglat A.A. (2020). Rapid Sonochemically-Assisted Synthesis of Highly Stable Gold Nanoparticles as Computed Tomography Contrast Agents. Appl. Sci..

[81] Yasuda K., Sato T., Asakura Y. (2020). Size-controlled synthesis of gold nanoparticles by ultrafine bubbles and pulsed ultrasound. Chem. Eng. Sci..

[82] Kumar P.S.S., Manivel A., Anandan S., Zhou M., Grieser F., Ashokkumar M. (2010). Sonochemical synthesis and characterization of gold–ruthenium bimetallic nanoparticles. Colloids Surf. A: Physicochem. Eng. Asp..

[83] Cui Y., Zhou D., Sui Z., Han B. (2014). Sonochemical Synthesis of Graphene Oxide-Wrapped Gold Nanoparticles Hybrid Materials: Visible Light Photocatalytic Activity. Chin. J. Chem..

[84] Wang L., Natan M., Zheng W., Zheng W., Liu S., Jacobi G., Perelshtein I., Gedanken A., Banin E., Jiang X. (2020). Small molecule-decorated gold nanoparticles for preparing antibiofilm fabrics. Nanoscale Adv..

[85] Yusof N.S.M., AshokKumar M. (2015). Sonochemical Synthesis of Gold Nanoparticles by Using High Intensity Focused Ultrasound. ChemPhysChem.

[86] Radziuk D., Grigoriev D., Zhang W., Su D., Möhwald H., Shchukin D. (2010). Ultrasound-Assisted Fusion of Preformed Gold Nanoparticles. J. Phys. Chem. C.

[87] Dheyab M.A., Aziz A.A., Jameel M.S., Khaniabadi P.M., Mehrdel B. (2021). Sonochemical-assisted synthesis of highly stable gold nanoparticles catalyst for decoloration of methylene blue dye. Inorg. Chem. Commun..

[88] Kaya E., Göksu H., Gerengi H., Arikan K., Ahamed M.I., Şen F. (2020). Magnetic Nanomaterials for Hydrogen Storage. Mater. Appl..

[89] Rajkumar S., Prabaharan M. (2019). Multi-functional core-shell Fe_3_O_4_@ Au nanoparticles for cancer diagnosis and therapy. Colloids Surf. B..

[90] Dheyab M.A., Aziz A.A., Jameel M.S., Abu Noqta O., Khaniabadi P.M., Mehrdel B. (2020). Simple rapid stabilization method through citric acid modification for magnetite nanoparticles. Sci. Rep..

[91] Khaniabadi P.M., Shahbazi-Gahrouei D., Aziz A.A., Dheyab M.A., Khaniabadi B.M., Mehrdel B., Jameel M.S. (2020). Trastuzumab conjugated porphyrin-superparamagnetic iron oxide nanoparticle: A potential PTT-MRI bimodal agent for herceptin positive breast cancer. Photodiagnosis Photodyn. Ther..

[92] Baskakov A., Solov’Eva A., Ioni Y.V., Starchikov S., Lyubutin I., Khodos I., Avilov A., Gubin S. (2017). Magnetic and interface properties of the core-shell Fe_3_O_4_/Au nanocomposites. Appl. Surf. Sci..

[93] Wang Z., Fu H., Tian Z., Han D., Gu F. (2016). Strong metal–support interaction in novel core–shell Au–CeO_2_ nanostructures induced by different pretreatment atmospheres and its influence on CO oxidation. Nanoscale.

[94] Wang X., Cui Y., Yu S., Zeng Q., Yang M. (2016). Core–shell interaction and its impact on the optical absorption of pure and doped core-shell CdSe/ZnSe nanoclusters. J. Chem. Phys..

[95] Kim Y.S., Lee S.M., Govindaiah P., Lee S.J., Lee S.H., Kim J.H., Cheong I.W. (2013). Multifunctional Fe_3_O_4_ nanoparticles-embedded poly (styrene)/poly (thiophene) core/shell composite particles. Synth. Met..

[96] Knopp D., Tang D., Niessner R. (2009). Bioanalytical applications of biomolecule-functionalized nanometer-sized doped silica particles. Anal. Chim. Acta.

[97] Kumar V.B., Annamanedi M., Prashad M.D., Arunasree K.M., Mastai Y., Gedanken A., Paik P. (2013). Synthesis of mesoporous SiO 2–ZnO nanocapsules: Encapsulation of small biomolecules for drugs and “SiOZO-plex” for gene delivery. J. Nanopart. Res..

[98] Mitsudome T., Kaneda K. (2013). Advanced Core-Shell Nanoparticle Catalysts for Efficient Organic Transformations. ChemCatChem.

[99] Pustovalov V., Astafyeva L., Fritzsche W. (2012). Optical properties of core–shell gold–silver and silver–gold nanoparticles for near UV and visible radiation wavelengths. Plasmonics.

[100] El-Toni A.M., Habila M.A., Labis J.P., Alothman Z.A., Alhoshan M., Elzatahry A.A., Zhang F. (2016). Design, synthesis and applications of core–shell, hollow core, and nanorattle multifunctional nanostructures. Nanoscale.

[101] Lin F.-H., Doong R.-A. (2011). Bifunctional Au−Fe_3_O_4_ Heterostructures for Magnetically Recyclable Catalysis of Nitrophenol Reduction. J. Phys. Chem. C.

[102] Xin Y., Fu-Bing X., Hong-Wei L., Feng W., Di-Zhao C., Zhao-Yang W. (2013). A novel H_2_O_2_ biosensor based on Fe_3_O_4_–Au magnetic nanoparticles coated horseradish peroxidase and graphene sheets–Nafion film modified screen-printed carbon electrode. Electrochim. Acta.

[103] Karamipour S., Sadjadi M., Farhadyar N. (2015). Fabrication and spectroscopic studies of folic acid-conjugated Fe_3_O_4_@Au core–shell for targeted drug delivery application. Spectrochim. Acta Part A Mol. Biomol. Spectrosc..

[104] Dheyab M.A., Aziz A.A., Jameel M.S., Khaniabadi P.M., Mehrdel B., Khaniabadi B.M. (2020). Gold-coated iron oxide nanoparticles as a potential photothermal therapy agent to enhance eradication of breast cancer cells. J. Phys. Conf. Ser..

[105] Li J., Zheng L., Cai H., Sun W., Shen M., Zhang G., Shi X. (2013). Facile One-Pot Synthesis of Fe_3_O_4_@Au Composite Nanoparticles for Dual-Mode MR/CT Imaging Applications. ACS Appl. Mater. Interfaces.

[106] Zhou T., Wu B., Xing D. (2012). Bio-modified Fe_3_O_4_core/Au shell nanoparticles for targeting and multimodal imaging of cancer cells. J. Mater. Chem..

[107] Smith M., McKeague M., DeRosa M.C. (2019). Synthesis, transfer, and characterization of core-shell gold-coated magnetic nanoparticles. MethodsX.

[108] Kostevšek N., Rožman K.Ž., Arshad M.S., Spreitzer M., Kobe S., Šturm S. (2015). Multimodal Hybrid FePt/SiO_2_/Au Nanoparticles for Nanomedical Applications: Combining Photothermal Stimulation and Manipulation with an External Magnetic Field. J. Phys. Chem. C.

[109] Dheyab M.A., Aziz A.A., Jameel M.S., Abu Noqta O., Khaniabadi P.M., Mehrdel B. (2020). Excellent relaxivity and X-ray attenuation combo properties of Fe_3_O_4_@Au CSNPs produced via Rapid sonochemical synthesis for MRI and CT imaging. Mater. Today Commun..

[110] Dheyab M.A., Aziz A.A., Jameel M.S. (2020). Synthesis and optimization of the sonochemical method for functionalizing gold shell on Fe_3_O_4_ core nanoparticles using response surface methodology. Surf. Interfaces.

[111] Brown K.R., Walter D.G., Natan M.J. (2000). Seeding of Colloidal Au Nanoparticle Solutions. 2. Improved Control of Particle Size and Shape. Chem. Mater..

[112] Liang R.-P., Yao G.-H., Fan L.-X., Qiu J.-D. (2012). Magnetic Fe_3_O_4_@Au composite-enhanced surface plasmon resonance for ultrasensitive detection of magnetic nanoparticle-enriched α-fetoprotein. Anal. Chim. Acta.

[113] Qiu J.-D., Peng H.-P., Liang R.-P., Xia X.-H. (2010). Facile preparation of magnetic core–shell Fe_3_O_4_@Au nanoparticle/myoglobin biofilm for direct electrochemistry. Biosens. Bioelectron..

[114] Wang L., Wang L., Luo J., Fan Q., Suzuki M., Suzuki I.S., Engelhard M.H., Lin Y., Kim N., Wang J.Q. (2005). Monodispersed core—shell Fe_3_O_4_@ Au nanoparticles. J. Phys. Chem. B..

[115] Hu Y., Meng L., Niu L., Lu Q. (2013). Facile Synthesis of Superparamagnetic Fe_3_O_4_@polyphosphazene@Au Shells for Magnetic Resonance Imaging and Photothermal Therapy. ACS Appl. Mater. Interfaces.

[116] Sodipo B.K., Aziz A.A., Mustapa M. (2015). Facile synthesis and characteristics of gold coated superparamagnetic iron oxide nanoparticles via sonication. Int. J. Nanoelectron. Mater..

[117] Wang L., Luo J., Maye M.M., Fan Q., Rendeng Q., Engelhard M.H., Wang C., Lin Y., Zhong C.-J. (2005). Iron oxide–gold core–shell nanoparticles and thin film assembly. J. Mater. Chem..

[118] Liang R.-P., Wang X.-N., Wang L., Qiu J.-D. (2014). Enantiomeric separation by microchip electrophoresis using bovine serum albumin conjugated magnetic core-shell Fe_3_O_4_@Au nanocomposites as stationary phase. Electrophoresis.

[119] Wani I.A., Ahmad T. (2013). Size and shape dependant antifungal activity of gold nanoparticles: A case study of Candida. Colloids Surf. B Biointerfaces.

[120] Jung W.-S., Park S.-H., Kadam A.N., Kim H., Lee S.-W. (2020). Direct hydrothermal synthesis of amine-functionalized cubic hematite (C-Fe_2_O_3_) and sonochemical deposition of nanosized Au for its application as a visible-light photocatalyst. Dalton Trans..

[121] Balachandramohan J., Anandan S., Sivasankar T. (2018). A simple approach for the sonochemical synthesis of Fe_3_O_4_-guargum nanocomposite and its catalytic reduction of p-nitroaniline. Ultrason. Sonochem..

[122] Okitsu K., Nunota Y. (2014). One-pot synthesis of gold nanorods via autocatalytic growth of sonochemically formed gold seeds: The effect of irradiation time on the formation of seeds and nanorods. Ultrason. Sonochem..

[123] Neto D.M.A., Freire R.M., Gallo J., Freire T.M., Queiroz D.C., Ricardo N.M.P.S., Vasconcelos I.F., Mele G., Carbone L., Mazzetto S.E. (2017). Rapid Sonochemical Approach Produces Functionalized Fe_3_O_4_ Nanoparticles with Excellent Magnetic, Colloidal, and Relaxivity Properties for MRI Application. J. Phys. Chem. C.

[124] Islam N., Van Phong L., Jeong J.-R., Kim C. (2011). A facile route to sonochemical synthesis of magnetic iron oxide (Fe_3_O_4_) nanoparticles. Thin Solid Films.

[125] Takahashi F., Yamamoto N., Todoriki M., Jin J. (2018). Sonochemical preparation of gold nanoparticles for sensitive colorimetric determination of nereistoxin insecticides in environmental samples. Talanta.

[126] Nagvenkar A.P., Perelshtein I., Piunno Y., Mantecca P., Gedanken A. (2019). Sonochemical One-Step Synthesis of Polymer-Capped Metal Oxide Nanocolloids: Antibacterial Activity and Cytotoxicity. ACS Omega.

[127] Mohammadi M.K., Hayati P., Jafari S., Karimi M., Gutierrez A. (2019). Sonication-assisted synthesis of a new rod-like metal-organic coordination polymer compound; novel precursor to produce pure phase nano-sized lead(II) oxide. J. Mol. Struct..

[128] Bagheri S., Aghaei H., Ghaedi M., Asfaram A., Monajemi M., Bazrafshan A.A. (2018). Synthesis of nanocomposites of iron oxide/gold (Fe_3_O_4_/Au) loaded on activated carbon and their application in water treatment by using sonochemistry: Optimization study. Ultrason. Sonochem..

